# A synthetic biology approach to self-regulatory recombinant protein production in *Escherichia coli*

**DOI:** 10.1186/1754-1611-6-2

**Published:** 2012-03-30

**Authors:** Martin Dragosits, Daniel Nicklas, Ilias Tagkopoulos

**Affiliations:** 1 UC Davis Genome Center, University of California, Davis, USA; 2 Department of Biomedical Engineering, University of California, Davis, USA; 3 Department of Computer Science, University of California, Davis, USA; 4 University of Natural Resources and Life Sciences, Vienna, Department of Chemistry, Vienna, Austria

**Keywords:** Synthetic biology, Recombinant protein, Self-regulatory, Escherichia coli, Stress promoter

## Abstract

**Background:**

Recombinant protein production is a process of great industrial interest, with products that range from pharmaceuticals to biofuels. Since high level production of recombinant protein imposes significant stress in the host organism, several methods have been developed over the years to optimize protein production. So far, these trial-and-error techniques have proved laborious and sensitive to process parameters, while there has been no attempt to address the problem by applying Synthetic Biology principles and methods, such as integration of standardized parts in novel synthetic circuits.

**Results:**

We present a novel self-regulatory protein production system that couples the control of recombinant protein production with a stress-induced, negative feedback mechanism. The synthetic circuit allows the down-regulation of recombinant protein expression through a stress-induced promoter. We used *E. coli *as the host organism, since it is widely used in recombinant processes. Our results show that the introduction of the self-regulatory circuit increases the soluble/insoluble ratio of recombinant protein at the expense of total protein yield. To further elucidate the dynamics of the system, we developed a computational model that is in agreement with the observed experimental data, and provides insight on the interplay between protein solubility and yield.

**Conclusion:**

Our work introduces the idea of a self-regulatory circuit for recombinant protein products, and paves the way for processes with reduced external control or monitoring needs. It demonstrates that the library of standard biological parts serves as a valuable resource for initial synthetic blocks that needs to be further refined to be successfully applied in practical problems of biotechnological significance. Finally, the development of a predictive model in conjunction with experimental validation facilitates a better understanding of the underlying dynamics and can be used as a guide to experimental design.

## Background

Recombinant or heterologous protein production (RPP) is an important biotechnological process, with applications that range from catalysis (e.g. washing detergents) and therapeutic use (e.g. antibody production), to protein production for enzymatic characterization and crystallography. Production of human proteins in bacteria dates back to the production of the 14-codon somatostatin gene in *Escherichia coli *in 1977 [1]. Since then, several hosts have been explored, including other prokaryote species [2], various yeast and fungal species [3], plant, insect, and mammalian cell lines [4]. As there is no universally optimal host, the choice of host is based on various parameters (protein yield, production time, etc.) on a case-by-case basis.

One of the most critical parameters, especially for proteins of therapeutic interest, is the presence of post-translational modifications. Complex proteins might harbor disulfide bonds as well as complex glycan structures (e.g. antibodies and antibody fragments) that influence the 3D structure, serum stability and the protein effector functions [5]. However, in the case of *E. coli*, many engineered strains and expression platforms were made available over the years [6], including strains that enable some complex post-translational modifications [7]. These advances, together with its easy cultivation, fast growth, and well-studied physiology explain *E. coli *'s role as a major host for RPP, including sensitive applications such as therapeutic protein production [8].

Over-expression of recombinant proteins can lead to significant stress in the host cell, which in turn limits its capacity to function as a cell factory. First, it constitutes a general metabolic burden, as it is responsible for depletion of precursor metabolites [9]. Recombinant proteins are usually produced in very high amounts (it is not unusual to comprise 30% or more of total cellular protein in the cell), which leads to a significant stress for the cell. The latter reacts by employing a heat-shock like response, which involves the induction of chaperones, foldases and proteases [10]. Another potential drawback of RPP in bacterial cells is that many recombinant proteins form inclusion bodies (IBs) which represent insoluble protein aggregates and necessitates elaborate downstream processing including de/re-naturation methods [8]. Recent work shows that IB-trapped proteins may actually be used directly [11], a result that contradicts the traditional thinking that IBs consist of misfolded, and thus inactive, protein. Still, in both cases further processing is needed to achieve soluble protein products [12]. On the other hand, the formation of inclusion bodies can also represent a favorable factor as the formation of such insoluble protein greatly facilitates initial protein enrichment. As such, the protein of interest as well as the purpose of the recombinant product will determine the desirable approach.

The goals of RPP are high protein yield and bioactivity, two variables that can have opposing dynamics. Despite notable advances (Table 1), the need for novel strategies that facilitate predictable and robust protein production processes is clearly present [13,14]. Most of the available expression platforms depend on either constitutive expression or fine-tuning of inducer concentrations, in order to adjust factors such as protein yield and solubility. For example, molecular chaperones and foldases are usually co-expressed from accessory plasmids with no further control [15]. However, imbalanced expression of these proteins can have detrimental effects on the production process. This leads to time-consuming fine-tuning, that is not robust to process modifications (e.g. a change in temperature or medium) as the optimal circuit operating point moves away from its previous value.

**Table 1:** Common techniques to optimize recombinant protein production in bacteria

Method	Characteristics
Host strain	Natural and engineered host strains can accommodate higher recombinant protein yields

Plasmid copy number	The choice of the plasmid backbone influences the production process through gene dosage

Inducer concentration	Inducer concentration influences transcription rate and therefore product formation/aggregation rate.

Promoter	Different promoters can be considered. Weak/Strong, constitutive and inducible promoters.

Ribosome binding site (RBS)	Position and sequence of the RBS influences translational efficiency

mRNA stability and structure	mRNA turnover influences the production process as well as mRNA structure can influence ribosome binding and translational efficiency

Codon optimization	Codon usage in the sequence of the recombinant gene greatly impacts translation efficiency

Process conditions	Temperature, oxygenation, pH and medium osmolarity impact on the production process

Medium composition	Optimization of the growth medium can lead to increase of the product yield and decrease of by-product formation

Heat shock protein co-overexpression and knockouts	Increased or decreased amount of several molecular chaperones, foldases and proteases influence protein yield and quality.

In depth review of these methods is available in [14]

In order to address challenges such as balancing protein production and cellular stress, we developed a synthetic expression platform that enables the cell to shut down the RPP mechanism by itself, once stress signals are detected. For this implementation we used and created new standardized parts and developed a computational model to elucidate the dynamics of this protein expression system. This study illustrates the potential of synthetic biology to help traditional biotechnological fields by constructing customized circuits with desired behaviors from standardized parts.

## Results and Discussion

### Overview and parts selection

**Figure 1:**
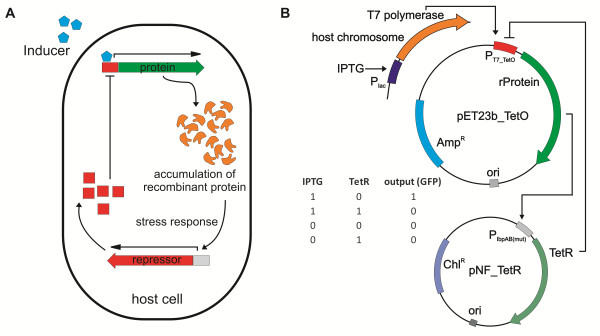
**A stress-limiting, self-regulatory protein production system**. **(A) **Schematic presentation of recombinant protein production coupled to a negative feedback mechanism in an *E. coli *host cell. **(B) **Experimental setup for the implementation of a negative feedback mechanism into a protein production system. A modified pET23(+) vector harboring a TetR binding site (pET23b_TetO) is used for recombinant protein (rProtein) expression. On a 2nd plasmid (pNF_TetR), TetR is expressed under the control of the stress sensitive P*IbpAB *promoter.

The general idea behind this new approach is to enable the cell to reduce the production of recombinant protein when significant cellular stress is detected (Figure 1A). The parts necessary for this self-regulated protein production system are (a) a flexible, repressible expression system, (b) a stress-induced promoter and (c) a suitable repression mechanism.

As shown in Figure 1B, we selected the pET expression system in combination with the *E. coli* C41 (DE3) strain, both widely used for laboratory scale protein expression [16]. The C41 strain encodes the T7 polymerase under the control of the *lac *promoter, and we cloned the recombinant protein downstream of a strongly-regulated T7 promoter. Addition of IPTG (Isopropyl β-D-1-thiogalactopyranoside) to the medium triggers the expression of T7 polymerase, which in turn transcribes the recombinant gene. Next, we identified a stress-sensitive promoter that is induced in recombinant protein production settings based on available literature. The *IbpAB *operon, which encodes inclusion-body binding proteins A and B [17], is known to be significantly up-regulated during the expression of various recombinant products [9,18-21], so we selected its promoter for the expression of a repressor protein. The TetR protein [22] was chosen as a repressor here due to the tight repression that it confers. To achieve transient repression for a relatively short time, we used a TetR protein with a degradation tag fusion [23] to decrease TetR protein half-life. We considered the application of repressor variant tagged for degradation necessary because we aimed at a relatively fast repressor turn-over. Previous research on synthetic gene circuits highlight, that otherwise such synthetic circuits suffer from a long response time [24,25], which may render them unsuitable for recombinant protein production processes. Finally, green fluorescence protein (GFP) was used as the model recombinant protein due to its high yields and capacity for rapid, inexpensive screening. Furthermore, the GFP mutant used in this study is known to form inclusion bodies, even when using weaker expression systems [26], which makes it a suitable model for evaluating the effect of a negative feedback circuit on protein production and solubility. For GFP expression experiments, saturating IPTG concentrations (1 mM) were applied.

### Integration of the Tet operator and stress promoter

**Figure 2:**
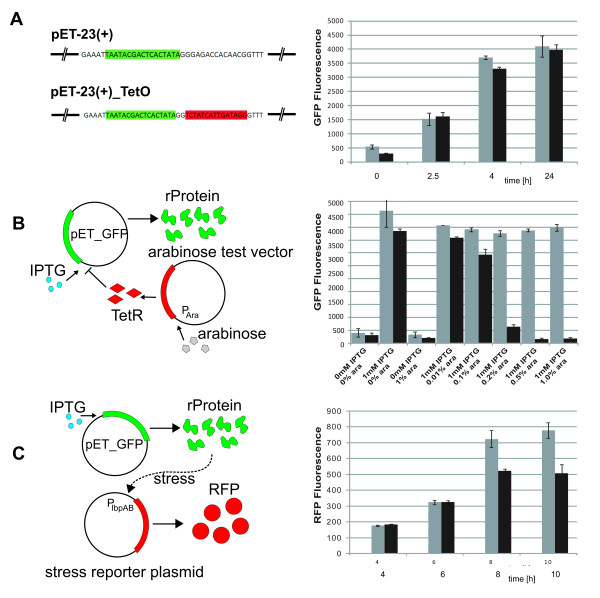
**Integration and testing of the circuit components**. **(A) **Integration of a TetO site downstream of the T7 promoter on pET-(23). Promoter highlighted in green and TetO site in red. The diagram depicts the expression of GFP from the pET-(23) vector (grey bars) and the TetO-site containing pET23b_TetO plasmid (black bars). **(B) **Effect of different arabinose concentrations on GFP expression in cells containing a plasmid encoding TetR under the control of the arabinose operon promoter and either the pET-(23) plasmid (grey bars) or pET-23_TetO plasmid (black bars) for GFP expression. %ara - arabinose concentration (% w/v). **(C) **Increased cellular stress in GFP expressing cells. Expression of GFP from pET23b_TetO and RFP expression using the P*IbpAB *on a 2nd accessory vector. RFP expression in cultures induced for GFP expression (grey bars) and non-induced cultures (black bars) is shown. All data represent averages of 4 biological replicates +/- standard error of the mean.

First, a Tet operator site (TetO) was inserted 2 bp downstream of the T7 promoter on the pET vector. Integration of the TetO has no impact to GFP production (GFP per OD 600) at the absence of TetR protein (Figure 2A). In contrast, when TetR was expressed using the arabinose operon promoter using a second plasmid, the GFP fluorescence levels decreased in a dose-dependent manner (Figure 2B). Previous research showed that the lactose and arabinose promoters show limited compatibility in terms of mutual regulation within a certain concentration range [27]. However, this subtle cross-regulation did not have an influence in the current experimental setup. To investigate the degree of induction for the wild-type (WT) stress-related *IbpAB *promoter (P*_IbpAB_*) by GFP production, we created a construct where the red fluorescence protein (RFP) was expressed under the control of P*_IbpAB _*(Figure 2C). We observed clear differential expression of RFP between cell populations induced and not-induced for GFP production.

### Feedback-based expression

**Figure 3:**
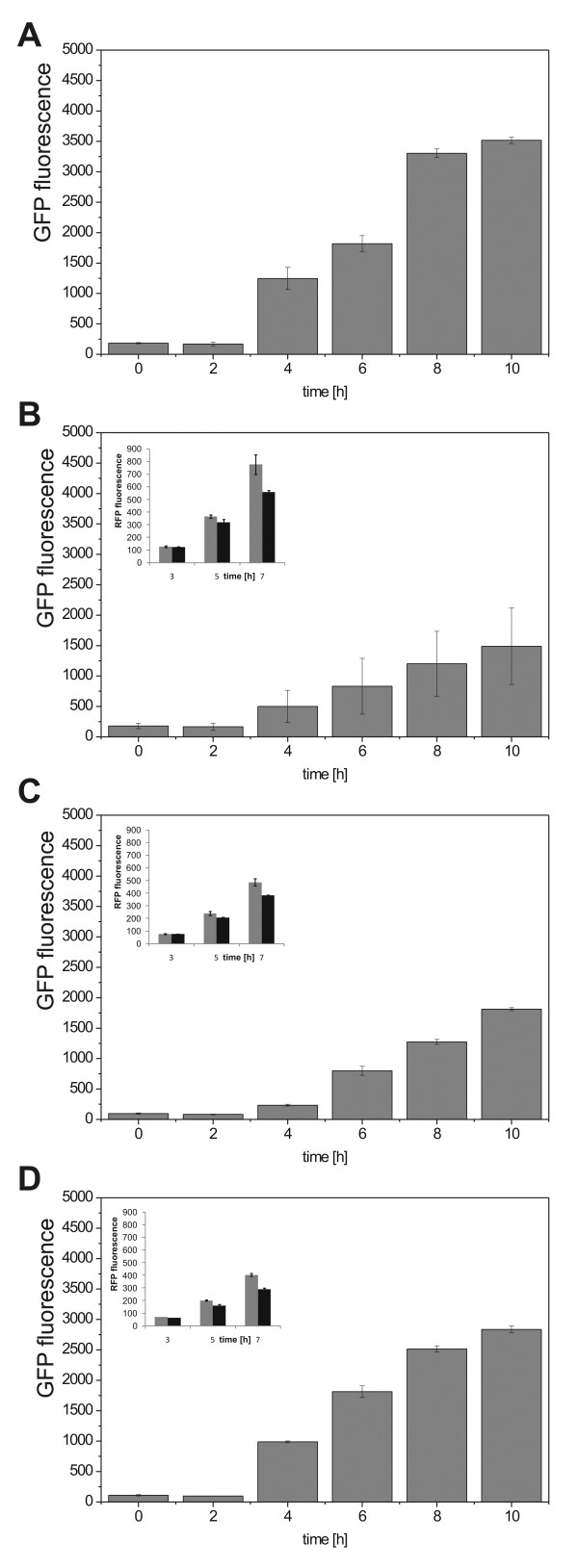
**GFP fluorescence in different expression system setups**. (**A**) without feedback system (**B**) WT *IbpAB *promoter and strong RBS for TetR expression (**C**) P*_I__bpA__B_* promoter mutant m3_2 and strong RBS and (**D**) P*_IbpAB_* promoter mutant m4_5 and weak RBS. Cultures were induced with 1 mM IPTG at 2 h. Insets in B-D show that GFP production triggers increased promoter activity for the WT and mutant promoters as indicated by increased RFP reporter expression (for the setup see Figure 2C). GFP expression has been normalized per OD600. Values represent averages +/- standard error of the mean.

Next, we completed the circuit by putting the *tetR *gene under the control of the wild-type P*_IbpAB _*promoter, resulting in plasmid pNF_TetR. Upon induction of the recombinant protein production circuit, we observed high reduction of fluorescence (GFP) levels (up to 70%) with respect to the control circuit where the feedback loop, encoded on pNF_TetR, is absent (Figure 3A). Interestingly, we observed a high variability of GFP expression across different clones, both in fluorescence levels and cell growth (Figure 3B, Additional file 1: Figure S1). Possible causes of the observed variability are subtle clone-specific differences in the cell's state, stochastic fluctuations, and non-controllable growth parameters during batch cultivation in shake flasks.

### Mutant libraries and dynamic range

The incorporation of the WT P*_IbpAB _*promoter led to high basal levels of promoter activity, significant decrease of intracellular GFP levels, and high variability in GFP expression. To increase the dynamic range of GFP expression that the circuit can operate and to investigate whether the variability and basal expression can be further reduced across cells, we created a P*_IbpAB _*mutant promoter library by using error-prone PCR that was characterized by using RFP as a reporter of promoter strength. A plasmid already containing the RFP gene was used to produce this mutant reporter library. Approximately 100 single clones of this library were analyzed with respect to RFP protein production and clones with reduced protein production with respect to the wild type P*_IbpAB _*promoter sequence were found. Sequencing elucidated the genetic basis of promoter variation, showing that 3 point mutations resulted in reduced expression in the 2 mutant promoters that were analyzed further. Furthermore, close proximity of the 3 nucleotide exchanges to the -35 region (promoter mutant m4_5) lead to stronger reduction of promoter activity (Additional file 1: Figure S2). We used RFP reporter constructs as described above in order to verify that the mutant promoters, despite lower basic activity, were still activated by recombinant protein stress which is typical for the *IbpAB *promoter (Figure 3C and 3D). Finally, those mutants were used to replace the original wild-type P*_IbpAB _*sequence in pNF_TetR. The application of these mutant promoter sequences lead to higher GFP levels and a lower clone-to-clone variability as compared with the negative feedback production system that uses the wildtype stress promoter for repressor production (Figure 3C).

For even higher flexibility, we engineered the ribosome binding site (RBS) to create an RBS library with mutants that have different (lower) levels of translational efficiency (Additional file 1: Table S3). Using lower translational efficiency RBS sequences in combination with promoter mutants for repressor expression, we were able to create a protein expression system that achieves comparable protein production levels with simultaneous induction of the stress-inducible system (Figure 3D). The delay that we observed in the production of the GFP is probably due to the basal activity of the *IbpAB *promoter.

### Influence of the stress feedback system on protein solubility

**Figure 4:**
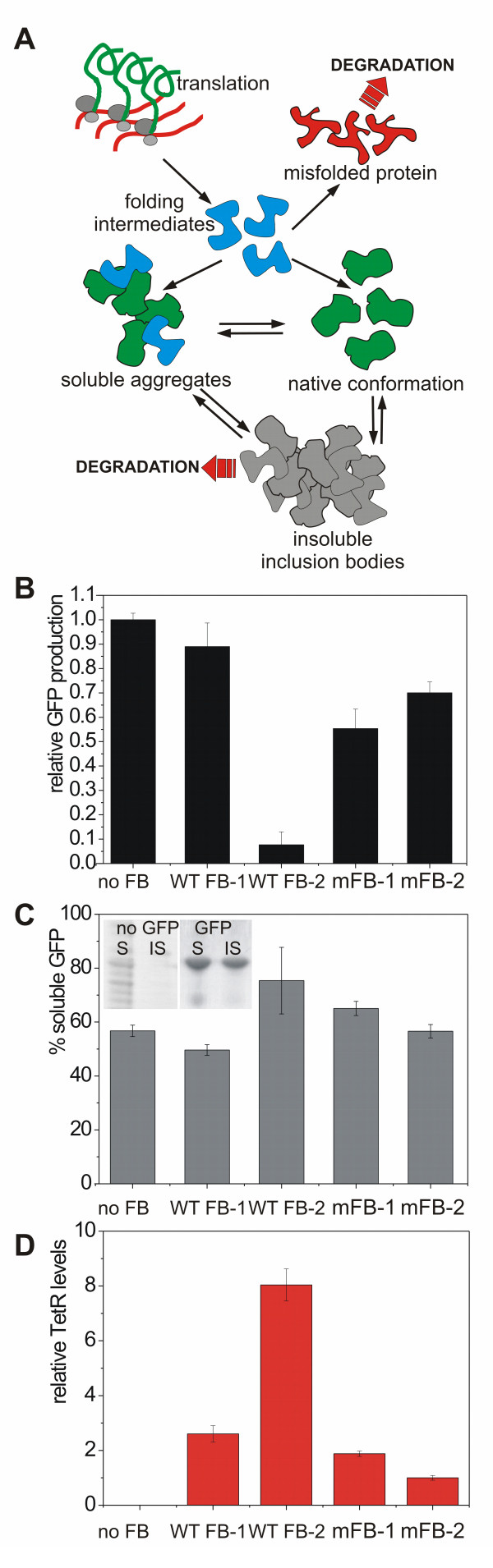
**Recombinant product and repressor production**. **(A) **Fate of recombinant proteins expressed in *E. coli*. After translation the folding intermediates of the protein can reach their native conformation, be deposited in soluble aggregates, or reach a misfolded state. Both native protein and soluble aggregates can be deposited in insoluble inclusions, from where polypeptides can be targeted for degradation. **(B) **Total amount of GFP (according to SDS PAGE) produced after 8 h of growth in different clones. Expression system without the use of pNF_TetR was set as internal reference (GFP content = 1) **(C) **Percentage of soluble GFP produced as a fraction of the total GFP. Insert in upper left corner depicts the difference in non-GFP producing and GFP producing cells in the soluble (S) and insoluble (IS) fraction. **(D) **Relative values of TetR protein in the soluble protein fraction according to Western Blot analysis. The mutant with the lowest TetR production (mFB-2) was set as reference to 1. No FB - no feedback plasmid used for GFP expression; WT FB-1 - set 1 of WT promoter clones; WT FB-2 - set 2 of wild type promoter clones (See Additional file 1: Figure S1 for further information); mFB-1 - mutant stress promoter 3_1 with strong RBS; mFB-2 mutant stress promoter and weak RBS. Values represent averages +/- standard error of the mean.

Inclusion body (IB) formation itself may represent an undesired process under several circumstances as described above and it readily occurs during recombinant protein expression in bacterial cells. Due to shortages in chaperone availability, a significant portion of the recombinant product may be deposited in these insoluble aggregates (Figure 4A). Since it is still unknown what percentage of active product is present in the IBs [11], we further analyzed the distribution of soluble and insoluble GFP for different promoter and RBS combinations. Induction of high level GFP expression as performed in the current study leads to the deposition of GFP in inclusion bodies (Figure 4C).

Our analysis shows that the native expression system without feedback leads to the highest amount of total GFP, but at the same time with a high percentage of insoluble GFP in the cell (Figure 4B and 4C). Some variants with the feedback-based expression system were found to increase the soluble fraction, albeit at the expense of lower protein yield. The observed dynamics are consistent with what we would expect due to the trade-off between protein solubility and yield. The reduced protein yield is expected as the feedback mechanism is designed to reduce GFP expression upon stress. Interestingly, variability across clones was also observed here, with some clones retaining the protein yield and quality of the native expression system, while others favoring protein solubility over protein yield, for the wild type P*_IbpAB _*promoter (with strong RBS) (Additional file 1: Figure S1). The intracellular levels of TetR protein were analyzed by Western blot analysis and support data obtained throughout the study showing that different variants and combinations of stress promoter and RBS indeed resulted in different intracellular TetR levels, with similar variability as in the case of GFP expression (Figure 4D).

### Effect of growth parameters on protein yield

**Figure 5:**
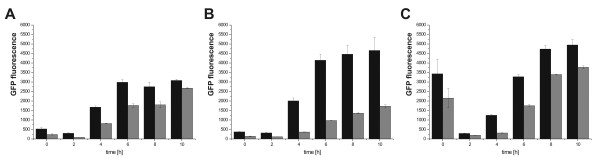
**Effect of growth parameters on circuit behavior**. Effect of medium composition on GFP expression in no-feedback (black) and weak feedback cells (promoter m4_5 and weak RBS, grey). GFP levels (Fluorescence per OD600) are shown. **(A) **M9 0.4% glucose **(B) **M9 0.4% glycerol and **(C) **LB medium. Values represent averages +/- standard error of the mean.

Since the small heat shock proteins *IbpA *and *IbpB *are part of the cellular protein folding machinery [10], their expression is not only limited to heat shock and recombinant protein stress but they rather exhibit a ubiquitous housekeeping function in maintaining protein stability and during protein turnover. This is consistent with our data as in some cases basal reporter production is present even in the absence of recombinant protein induced stress. In this context, factors such as media composition, growth rate and overall status of the bacterial culture may influence the behavior and performance of the system. To investigate the degree that our system is sensitive to media composition, we used glucose, glycerol or LB as growth media. Our results show that GFP production levels were clearly reduced for clones where the negative feedback system was integrated compared with expression clones that did not use the feedback system. In both cases, when the carbon source was changed from glucose to glycerol and when complex (LB) medium was used, the discrepancy between feedback and non-feedback GFP production was higher than on M9 glucose (Figure 5). Additionally, the status of the starter culture influenced the expression system during batch growth. Whereas starter culture growth medium had no effect on GFP fluorescence accumulation in the native expression system, in the stress-feedback system we observed that when starter cultures were grown on LB medium a delayed accumulation occurred compared with starter cultures grown on M9 medium (Additional file 1: Figure S3). One possible explanation is that because of the higher growth rate in LB, increased protein translation leads to increased P*_IbpAB _*activity. When different media were used for starter cultures, cells also entered the stationary phase at different time points, which is in agreement with our initial measurements that showed increased P*_IbpAB _*activity through transition to the stationary phase.

### Mathematical model

The production of recombinant product was modeled by dividing the process into three main sub-components: (a) the induction of T7 polymerase production by IPTG, (b) the recombinant protein production via the T7 promoter and (c) the stress-induced expression of the TetR repressor. A system of delayed differential equations was used to capture the transcription and translation processes for T7 polymerase, GFP and TetR. In addition, the ratio between GFP fractions was also modeled and visualized. A detailed mathematical description of the model, together with all experimentally derived and estimated parameters, is given in the supplementary materials section. We performed sensitivity analysis of our model (Additional file 1: Figure S4) that identified two key processes that are crucial for its robustness. In addition, we performed cross-validation to avoid over-fitting and evaluate the generalization error of the model (Additional file 1: Table S9).

**Figure 6:**
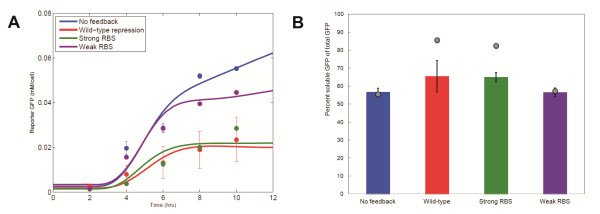
**Mathematical model of the protein production process**. **(A) **Time course simulation results for total GFP production for the no-feedback and three feedback systems over 12 hours. Points represent experimentally measured values. **(B) **Predicted and experimentally measured soluble GFP fractions after 8 hours for feedback mechanisms with different strength (color map as in 6A). Points represent simulation results. Experimental data are averages over four replicates, where bars +/- standard error of the mean.

As shown in Figure 6A, the model's predictions of the final protein concentrations and their dynamic profiles are consistent with the experimental data, with deviations that are attributed to inexact parameter estimation and simplifying assumptions in its description. In our analysis, the model was particularly useful in identifying the dynamic range of each participating compound. The model predicted a higher fraction of soluble product for cells with the self-regulatory mechanism present (Figure 6B). The difference between the experimentally measured and computationally derived values may stem from the fact that in the model we don't account for the effect of TetR proteins in the depletion of the cellular resources and folding machinery. Possible high-order effects, such as the formation of inclusion bodies that may actually increase the rate at which proteins enter their insoluble state, can also partially explain this discrepancy.

## Conclusion

In our quest to create self-regulatory systems for recombinant protein production, we used an integrated synthetic biology approach to construct a synthetic circuit that limits recombinant protein production through stress-induced feedback. We validated the functionality of different variants of the synthetic circuit in their capacity to limit stressful protein production, and to increase the total soluble fraction. Since the protein yield was significantly lowered in the process, further investigation on promoter and repressor engineering for avoiding such loss would be welcomed. In addition, since different proteins lead to different levels of stress within the host cells [9], it would be interesting to test this approach with other recombinant protein species. Furthermore different inducer concentrations can be used to tune transcription rate and product formation, although there is evidence that inducer concentration does not necessarily influence the formation of active soluble protein [28].

The computational model that we constructed provided valid predictions on the system dynamics, and was useful as a first order guiding tool for our experimental design. An extended phenomenological description and inclusion of a larger set of measured experimental parameters would allow an increased predictive accuracy of the model, and it may help to test or generate alternative hypotheses regarding the the dynamics of inclusion body formation and their degradation rates. This study provides an example of how integration of computational, engineering and experimental methods, together with the synthetic biology concepts of parts standardization, can be applied to address biotechnological challenges from a new perspective. Altogether, this and similar future studies can be applied to guide the construction of robust auto-regulatory protein production systems.

## Methods

### Host strains and growth media

*E. coli *DH5α was used for all cloning procedures, whereas *E. coli *C41 [29] was used as host expression strain. All cultivations were performed on LB medium and M9 minimal medium (0.4% w/v glucose) supplemented with antibiotics (carbenicillin 100 μg mL^-1 ^and chloramphenicol 25 μg mL^-1^) when necessary and incubated at 37°C and 150 rpm on an orbital shaker. For experiments involving GFP expression and synthetic circuit characterizations, cultures were grown on M9 medium and induced with 1 mM IPTG (1 M 0.22 μm filtered sterile stock solution). Cultures were inoculated at an optical density (600 nm) of 0.1 and grown for 2 h before induction with IPTG. For experiments involving arabinose as inducer substance, arabinose was added to the growth medium in a concentration range of 0-1% (from a 20% w/v 0.22 μm filtered sterile stock solution). Samples were taken in regular intervals to monitor growth and product formation. All tests involved at least 3 biological replicates and were performed in 5 mL growth medium and 50 mL tubes unless stated otherwise.

### Molecular cloning procedures

For standard molecular cloning restriction enzymes were purchased from New England Biolabs Inc. Taq polyermase (Qiagen) was used for screening and error prone PCR whereas other PCR reactions were performed using PfuTurbo proofreading polymerase (Stratagene). The native DNA sequence of P*_IbpAB _*was amplified from a genomic DNA isolation of *E. coli *MG1655. Subsequent error prone PCR was essentially performed as described previously [27]. To achieve higher error rates during PCR, imbalanced dNTPs (0.2 mMdATP and GTP, 1 mMdCTP and dTTP) and increased MgCl_2 _(20 mM) and MnCl_2 _(0-0.5 mM) concentrations were applied. Modification of ribosome binding sites was performed by mismatch oligonucleotide PCR. Plasmid mini prep DNA was used as template for PCR with primers harboring the desired nucleotide mutations of the RBS. After PCR parental plasmid was digested with the methylation sensitive restriction enzyme DpnI and linear mutated plasmid DNA was transformed into *E. coli *DH5α. Plasmid DNA from single colonies was isolated and sequenced in order to select mutated sequences.

Primer sequences and further information on cloning procedures as well as standard parts and newly constructed DNA parts used in this study can be found in Supplementary Online Materials (Text and Additional file 1: Table S1, Table S2 and Table S3, respectively).

### OD600 measurements and fluorescence measurements

Optical density, as a measure of cell mass, was determined on a Biophotometer (Biorad) and/or an Infinite M200 plate reader (Tecan). Fluorescence measurements of GFP were performed on an Infinite M200 microtiter-plate reader (Tecan) using the following settings: 37°C, excitation wavelength 485 nm, emission wavelength 535 nm and gain 75. GFP fluorescence values in the manuscript are given as GFP fluorescence per OD600.

### Analysis of soluble and insoluble proteins and western blotting

Preparations of soluble and insoluble protein were performed according to the pET vector manual (Novagen, pET system manual, 11^th ^edition, 2006) with small modifications. In short, cells were harvested by centrifugation, wet cell weight was determined and samples were immediately frozen at -20°C for at least 24 h. For protein analysis, samples were thawed to room temperature and re-suspended in cell lysis buffer. Lysozyme (Sigma) was added (60 kU per g wet biomass) and samples were incubated at room for 45 min on a rotary shaker. To complete cell lysis and decrease the viscosity of the solution, samples were treated in a Bioruptor sonication apparatus (Diagenode) at high power setting and a 30s sonication interval for 10 min. After centrifugation the supernatant containing soluble proteins was separated from insoluble cell fraction. The pellet was re-suspended in cell lysis buffer containing 1% SDS and kept as insoluble protein fraction. SDS PAGE analysis was performed using 12% BisTris polyacrylamide Novex gels (Invitrogen) according to standard protocols. Gels were stained with PageBlue staining solution (Thermo Scientific). One sample was loaded on all of the gels to account and correct for variations in gel staining. After scanning of gels, protein bands were quantified using GelAnalyzer v2010a http://www.gelanalyzer.com/. Intensity of gel bands was corrected for staining differences between individual gels and normalized to the wet cell weight of each sample to account for the different initial sample volumes.

Intracellular levels of TetR protein were determined by Western Blotting. Total soluble protein was separated by standard SDS-PAGE as described and blotted onto a PVDF membrane. After membrane blocking (TBS 0.1% Tween-20, 1% milk protein) at 4°C over night, TetR protein was detected using an anti-TetR polyclonal rabbit antibody serum (Thermo Scientific), a polyclonal anti-rabbit Peroxidase conjugate (Sigma Aldrich) and a BCL plus detection kit (GE Healthcare) using standard WB protocols. Western Blot signals were acquired on a Storm scanner (GE Healthcare) at 520 nm and analyzed using ImageQuant image analysis software (GE Healthcare). Normalization of TetR intensities on the blots was performed as described for GFP levels above.

### Mathematical model

The mathematical four replicates, where bars equations that incorporate the specific time required for particular events, such as transcription and translation. The equations were simulated through the "dde23" routine in MATLAB 7.10 (The MathWorks, Natick, MA). All results are displayed for each species over 12 hours of batch culture with induction at two hours. The system was allowed reach steady state prior to *t *= 0 hours for an extended period of time. During this pre-induction phase, the growth rate was set to *μ_0_*. Simulation results were obtained under induction by 1 mM of IPTG. In order to compare the model's single cell results, experimental data was converted from normalized fluorescence to molarity [30]. Detailed transcription of the model can be found in Supplementary Material online.

## Competing interests

The authors declare that they have no competing interests.

## Authors' contributions

MD performed the experimental work, DN developed the mathematical model, IT supervised experimental and computational procedures, MD, DN, and IT analyzed the data and wrote the manuscript. All authors read and approved the final manuscript.

## Supplementary Material

Additional file 1 **The additional pdf file hosts detailed description of the computational methods, biological parts numbers, as well as supplemental additional figures and tables and can be retrieved online as Additional Material File**. Click here for file
