# Supplementary material for: A synthetic biology approach to self-regulatory recombinant protein production in Escherichia coli
Source: J Biol Eng. 2012 Mar 30;6:2. doi: 10.1186/1754-1611-6-2 (PMC3384244; doi:10.1186/1754-1611-6-2)
Supplement: Additional file 1 — The additional pdf file hosts detailed description of the computational methods, biological parts numbers, as well as supplemental additional figures and tables and can be retrieved online as Additional Material File. [file 1754-1611-6-2-S1.PDF]

A synthetic biology approach to self-regulatory recombinant protein production in *Escherichia coli*

Martin Dragosits[1†], Daniel Nicklas[1, 2], Ilias Tagkopoulos[1, 3*]

Supplementary Online Materials

e-mail:

martin.dragosits@boku.ac.at

denicklas@ucdavis.edu

iliast@ucdavis.edu

[1] UC Davis Genome Center, University of California, Davis

[2] Department of Biomedical Engineering, University of California, Davis

[3] Department of Computer Science, University of California, Davis

[†] Current address: University of Natural Resources and Life Sciences, Vienna, Department of Chemistry, Vienna, Austria

* Corresponding author contact information: Ilias Tagkopoulos, Department of Computer Science, One Shields Avenue, University of California, Davis, 95616, Davis, USA. Fax: +1 (530) 752-4767 Tel: +1 (530) 752-7707

1. Biological Parts

All parts were obtained from the Registry of Standard Biological Parts (RSBP), with the exception of pET-23b, that was obtained from EMD biosciences (Table S1).

GFP expression using pET-23 and pET-23 derivate: GFPmut3 was obtained from the RSBP and cloned into the expression vector to obtain intracellular expression by removing the PelB leader sequence from the vector backbone.

In order to create the TetR expression system we used the biobrick part encoding for RBS, gene sequence and terminator by amplifying the whole cassette using biobricks compatible primers with standard restriction sites. For the amplification of P_{IbpAB} from genomic *E. coli* MG1655 DNA, also biobrick compatible primers were used. Initial experiments involving TetR expression using the arabinose operon promoter (P_{Ara}) were done using pSB1C3 as plasmid backbone which was later on replaced by plasmid pSB3C5 (p15A origin of replication) for co-maintenance in cells harboring a pET plasmid (ColE1 origin of replication) due to plasmid compatibility issues.

Primers used in this study for molecular cloning are listed in Table S2. For plasmid/insert sequencing, standard DNA oligos (binding on the vector backbone) were used.

2. Computational Model

We have developed a mathematical model that tracks the concentrations of each key species over the desired time course. The mathematical descriptions are built on a system of delay differential equations (DDEs) that incorporate the specific time required for particular events, such as transcription and translation. Table S4 contains the full system of equations. To best describe the overall system, we divide it into its primary components: induction and recombinant protein production.

Induction Model: Equations (3)-(5) track induction with IPTG to the subsequent production of T7 RNA polymerase (T7 RNAp). In Tables S5 and S6, we have included a description of each parameter found in these equations. As a first step, the batch culture of cells is allowed to grow for a given period of time before saturating (1mM) IPTG (*I*) is added to the extracellular media. At this point, the compound must traverse across the cell membrane, which may be described through an ordinary differential equation [1]. In equation (3), μ represents the growth rate, diluting the IPTG concentration due to cell division. This is equivalently included in the expression for each species. In order to properly account for cell growth, we fit a sigmoidal curve of the following form to experimental data for each repressor strength.

$$M = \frac{1}{1 + \exp(-at + b)}, \tag{1}$$

Where *M* represents the number of cells at a given time *t*, while *a* and *b* are constants determined by logistic regression. The time derivative of equation (1) gives μ, which is evaluated at each time point during the numerical integration.

Once IPTG enters the cell, it may reversibly bind to LacI, allowing for the production of T7 RNAp mRNA. To keep track of total T7 RNAp presence and maintain mechanistic detail, we break this up into its respective transcribed mRNA (T_m) and translated protein (T) concentrations. The resulting value of the latter will lead to GFP production at an exceedingly rapid rate. As mentioned earlier, these types of events take a specific amount of time before mRNA or protein is created. This delay is captured in two ways: allowing the rate of change to be dependent on previous values of inducer or mRNA concentrations and including a dilution decay term while this takes place.

To accomplish this, we incorporate an in-depth model of the *lac* operon developed by Yildirim & Mackey [2] that emphasizes the key time delay prior to these events. To do so, they have utilized a system of DDEs along with the operator-repressor mechanics inherent to the natural system, which this recombinant circuit takes advantage of in order to produce T7 RNAp. We assume that many of the properties of the natural system are carried over to the recombinant circuit, such as the operator dynamics and the steady value of mRNA and protein. They begin with a previously described general form to describe the fraction of free operators:

$$f(I) = \frac{1 + K_1 I^n}{K_2 + K_1 I^n}, \tag{2}$$

where I is the inducer, which is allolactose for the wild-type system, but corresponds to IPTG concentration here. K_1 and K_{op} (not shown above) are equilibrium constants for inducer-repressor and repressor-operator reactions, respectively. K_2 represents the value of $1 + K_{op}R_{tot}$, where R_{tot} equals the total amount of repressor. Finally, n corresponds to a Hill coefficient appropriate for the system. Maximum transcription occurs when I approaches infinity and the system is fully induced. Transcription will proceed with no inducer in the system, but is modulated by a factor of K_2.

With the general dynamics of transcription described, we can now look at the specific delay differential equation. In equation (4), $\tilde{I}$ represents the concentration of IPTG at a particular time ago. This is the introduction of delay components and can be written out fully as $I(t - \tau_{m7})$, where τ_{m7} equals the amount of time it takes for native RNA polymerase to transcribe the gene encoding T7 RNAp. Henceforth, this notation is maintained, but note that each species has a characteristic time and the associated value can be found in table S7. In conjunction with this delay, the exponential term incorporates the dilution of inducer that will take place due to growth during that interval.

The initial rate term, k_{m7} is derived from the steady state condition without induction. Yildirim & Mackey [2] report that initial *E. coli* RNA levels sit at 2.08e-6 mM, which we use as the initial value of T7 RNAp mRNA. From this, we have

$$k_{m7} = k_{2tm}(2.08 \times 10^{-6} \text{ mM})\left(\mu_0 + k_{degM}\right),$$

Where μ_0 is the initial growth rate, derived by evaluating the time derivative of equation (2) at $t = 0$ hours. The remaining terms in equation (4) are adapted from equation (2).

With this, we can now display the simpler differential equation for T7 RNA polymerase protein production. Equation (5) assumes that production of the protein is proportional to the concentration of T7 mRNA at a time τ_{p7} hours ago, the duration of the translation event. Similar to equation (4), the concentration of mRNA is diluted during this time, requiring the

exponential term. The final parameter, k_{p7}, is derived in an identical fashion as k_{m7} with the understanding that in non-induced steady state, a protein under control by the *lac* operon will be present at a concentration of roughly 20 molecules/cell [2]. This results in 20 times more T7 RNAp protein than T7 RNAp mRNA in the absence of inducer, providing an initial value for this protein. As a mathematical relation, we have

$$k_{p7} = 20(\mu_0 + k_{degT})e^{\mu_0 \tau_{p7}}$$

We have now completed the induction model as the system proceeds to produce recombinant protein.

Recombinant Protein Production Model: Similar to T7 RNAp production, formation of GFP, the reporter protein, is divided into its respective transcription (G_m) and translation (G) product. For simplicity, the model allows translation only into folded GFP, which may subsequently misfold (G_i) and contribute to inclusion body aggregation.

Equations (6)-(9) outline this process and a description of each parameter is found in table S6. With little evidence of misfolded GFP degradation kinetics, we have assumed it equal to that of the natural reporter protein. Equation (6) is governed by two production components and the canonical degradation/dilution term. First, we need to determine the amount of free operator sites in an identical fashion as equation (2). Since there is no inducer to block TetR from the TetO site in this system, we take $I = 0$. Clearly, without TetR, transcription will proceed at its maximal rate as the fraction reduces to one. The second part utilizes Michaelis-Menten kinetics to characterize the rate of transcription by T7 RNAp [3]. A general form for the rate (v) is as follows:

$$v = \frac{k_{cat}ES}{K_m + S},$$

where K_m represents the Michaelis constant and k_{cat} is the maximal rate of transcription by polymerase E acting on DNA strand S. These are replaced with k_{2gm} and k_{3gm} for k_{cat} and K_m, respectively. Similarly, E is equivalent to T, while S becomes C_{vect}, the concentration of plasmid vector in each cell. For the pET expression system, the copy number takes an

average of 35 [4], yielding a concentration of 7.27e-5 mM/cell when approximating the volume of an *E. coli* cell as 8.00e-16 L [2].

Similar to k_{m7} and k_{p7}, we derive k_{pG} from steady state analysis without induction. Using equation (6) with no feedback, we can first calculate the steady state GFP mRNA value using 4.16e-5 mM of T7 RNAp under these conditions [2]. With this, k_{pG} is a straightforward calculation from equation (7) at steady state:

$$k_{pG} = \frac{k_{Gi}(\bar{G})^{n_{Gi}} + \left(k_{degG} + \mu_0\right)\bar{G}}{\overline{G_m}} e^{\mu_0 \tau_{pG}},$$

where overbars dictate steady state values and $\bar{G} = 2.67\text{e-}3$ mM from experimental data at $t = 2$ hours.

Finally, we can calculate k_{Gi} by utilizing SDS-PAGE data for folded and misfolded GFP as $t \to \infty$ after induction.

$$k_{Gi} = k_{degG}\left[\frac{G_{i,t\to\infty}}{(G_{t\to\infty})^{n_{Gi}}}\right]$$

The key unknown dynamics in this model are those governing TetR production, characterized by k_{1r}, n_R, and τ_R. The former is a broad first-order generalization for a likely mathematically complex process, while the latter two describe the associated Hill coefficient and delay in TetR production, respectively. These were treated as free parameters in order to achieve a realistic match to experimental data.

Optimization was based on least squares matching to experimental results. Parameter sweeps were performed in order to minimize the error, *S*, given by

$$S = \sum_{i=1}^{N}\left(C(t_i) - y(t_i)\right)^2,$$

where $C(t_i)$ denotes the concentration of the optimized species and $y(t_i)$ gives the data point at that time. We sum over all time points in a given experiment and compare to the simulation results with a set of parameters. The variables are then altered independently and follow the appropriate gradient to minimize *S*. We have optimized entirely unknown parameters and

those with high sensitivity or values with which we had low confidence in their accuracy (Tables S7 and S8).

Results and Discussion

The equations were simulated through the "dde23" routine in MATLAB 7.10 (The MathWorks, Natick, MA). All results are displayed for each species over 12 hours of batch culture with induction at two hours. The system was allowed reach steady state prior to $t = 0$ hours for an extended period of time. During this pre-induction phase, the growth rate was set to μ_0. Simulations were performed under induction by 1 mM of IPTG. In order to compare the model's single cell results, experimental data was converted from normalized fluorescence to molarity [5].

In order to determine the key parameters in the entire process, we perform a sensitivity analysis. The system was perturbed by 1% in both directions to visualize the associated effect each variable has on steady state reporter GFP concentration. Scaled sensitivity coefficients were calculated as

$$\epsilon_{k_i} = \frac{\partial C}{\partial k_i}\left(\frac{k_i}{C}\right),$$

where C is the steady state GFP concentration and k_i represents each of the rate constants or parameters in the model. Significantly, the results of this analysis reveal a high sensitivity to both GFP misfolding and TetR production, highlighting the role of these processes in GFP production (Figure S4). Conversely, the system is substantially robust to perturbations in its delay parameters. Finally, in an effort to examine the system's optimization stability, we systematically remove one data point and re-optimize the estimated parameters for each feedback type. To compare the resulting optimization, we calculate the squared error of the solution for G with the initial and final parameter sets. These results are displayed in Table S9.

References

1. Miao F, Kompala DS: **Overexpression of cloned genes using recombinant Escherichia coli regulated by a T7 promoter.***Biotechnol and Bioeng* 1992, **40**(7):787-796.
2. Yildirim N, Mackey M: **Feedback regulation in the lactose operon: a mathematical modeling study and comparison with experimental data.***Biophys J*2003, **84**(5):2841-2851.
3. Takinoue M, Kiga D, Shohda K, Suyama A: **Design and numerical analysis of RNA oscillator.***Proceedings in Information and Communications* 2009, 201-212.
4. Novagen: **pET System Manual**. 2003.
5. Registry of Standard Biological Parts: **Part:Bba_E0040**, 2004.
6. Barkley MD, Riggs AD, Jobe A, Bourgeois S: **Interaction of effecting ligands with Lac repressor and repressor-operator complex.** *Biochemistry* 1975, **14**(8): 1700-1712.
7. Kędracka-Krok S, Wasylewski Z: **Kinetics and equilibrium studies of Tet repressor-operator interaction.** *Journal of Protein Chemistry* 1999, **18**(1): 117-125.
8. Martin CT, Coleman JE: **Kinetic analysis of T7 RNA polymerase-promoter interactions with small synthetic promoters.***Biochemistry*1987, **26**: 2690-2696.
9. Bernstein JA, Khodursky AB, Lin PH, Lin-Chao S, Cohen SN: **Global analysis of mRNA decay and abundance in Escherichia coli at single-gene resolution using two-color fluorescent DNA microarrays.***P Natl Acad Sci USA*2002, **99**(15): 9697-9702.
10. Arnold S, Sieman M: **Kinetic modeling and simulation of in vitro transcription by phage T7 RNA polymerase.** *Biotechnol and Bioeng* 2000, **72**(5): 548-561.
11. Becskei A, Serrano L: **Engineering stability in gene networks by autoregulation.** *Nature* 2000, **405**(6786): 590-593.

3. Supplementary Tables

part	supplier	part no#
pET-23b(+)	EMD biosciences	69746
GFPmut3 w/o tag	RSBP	E0040
TetR with degradation tag	RSBP	C0040
Arabinose promoter	RSBP	I0500
mRFP	RSBP	E1010
pSB1C3	RSBP	pSB1C3
RBS	RSBP	B0034
RBS	RSBP	B0031
RBS	RSBP	B0064
Terminator sequence	RSBP	B0015
pSB3C5	RSBP	pSB3C5

Table S1. DNA Parts used in this study to create a negative feedback system.

oligo name	5' 3' sequence	Used for
GFPmut3_fw	agcatatgcgtaaaggagaagaact	GFP cloning pET23b
GFPmut3_rv	ttgaattcttattattatttgtatagttcatcca	GFP cloning pET23b
GFPmut3_fw	atatattaatatgcgtaaaggagaagaact	GFP cloning pET23b
T7_tetO	gaagatctcgatcccgcgaaattaatacgactcactataggtctatcattgatagggtttccctctagata	modified T7 with TetO
T7_fw	gaagatctcgatcccg	fw for modified T7
T7_TetO_rv	tatctagagggaaaccc	bw for modified T7
PIbpAB_fw	ctgcagcggccgctactagtaatcggctacgatgtaaaaatgggtctggaa	cloning of IbpAB promoter
PIbpAB_rv	gaattcgcggccgcttctagagattcatctgttgatcgtgggtgttggcct	cloning of IbpAB promoter
mRFP_fw	gaattcgcggccgcttctagagtactagagaaagaggagaaatactagatgg	cloning of mRFP into pSB1C3_PIbpAB
mRFP_rv	ctgcagcggccgctactagtatataaacgcagaaaggcccacccgaa	cloning of mRFP into pSB1C3_PIbpAB
RBSver31_fw	cgattactagatcacacaggaaacctactagatgtc	mutation RBS on pSB3C5
RBSver31_rv	gacatctagtaggtttcctgtgtgatctagtaatcg	mutation RBS on pSB3C5
RBSver64_fw	cgattactagaaaagaggggaaatactagatgtc	mutation RBS on pSB3C5
RBSver64_bw	gacatctagtatttcccctcttttctagtaatcg	mutation RBS on pSB3C5

Table S2. DNA primers used for cloning procedures

RSBP number	sequence	Relative activity
B0034	TCTAGAGAAAGAGGAGAAATACTAGATG	1
B0031	TCTAGAGTCACACAGGAAACCTACTAGATG	0.07
B0064	TCTAGAGAAAGAGGGGAAATACTAGATG	0.35

Table S3. Ribosome binding sites with different strength according to the RSBP. B0034 was the original RBS which was replaced by B0031 and B0064 to decrease translational efficiency.

$$\frac{dI}{dt} = A\left(\frac{I_0}{B + I_0}\right)\left(\frac{\mu}{k_{sat} + \mu}\right) - \mu I \tag{3}$$

$$\frac{dT_m}{dt} = k_{m7}\frac{1 + k_{1tm}(\tilde{I}e^{-\mu\tau_{m7}})^{n_T}}{k_{2tm} + k_{1tm}(\tilde{I}e^{-\mu\tau_{m7}})^{n_T}} - (\mu + k_{degM})T_m \tag{4}$$

$$\frac{dT}{dt} = k_{p7}\widetilde{T_m}e^{-\mu\tau_{p7}} - (\mu + k_{degT})T \tag{5}$$

$$\frac{dG_m}{dt} = \left(\frac{1}{1 + k_{1gm}\tilde{R}}\right)\left(\frac{k_{2gm}\tilde{T}e^{-\mu\tau_{mG}}C_{vect}}{k_{3gm} + C_{vect}}\right) - (\mu + k_{degM})G_m \tag{6}$$

$$\frac{dG}{dt} = k_{pG}\widetilde{G_m}e^{-\mu\tau_{pG}} - k_{Gi}(G)^{n_{Gi}} - (\mu + k_{degG})G \tag{7}$$

$$\frac{dG_i}{dt} = k_{Gi}(G)^{n_{Gi}} - (\mu + k_{degG})G_i \tag{8}$$

$$\frac{dG}{dt} = k_{1r}(\tilde{G})^{n_R}e^{-\mu\tau_R} - (\mu + k_{degG})R \tag{9}$$

Table S4. The system of delay differential equations (DDE) that was used in our mathematical model.

Type	Description	Type	Description
I_0	Constant extracellular IPTG concentration	k_{p7}	Maximum rate of T7 RNAp production
A	pH-dependent IPTG shuttling constant	k_{degM}	Degradation rate of mRNA
B	pH-dependent IPTG shuttling constant	k_{degT}	Degradation rate of T7 RNAp protein
k_{sat}	IPTG saturation value	n_T	Hill coefficient of T7 RNAp mRNA production
k_{m7}	Maximum rate of T7 RNAp mRNA production	τ_{m7}	Duration of T7 RNAp transcription
k_{1tm}	Equilibrium constant for IPTG-LacI binding	τ_{p7}	Duration of T7 RNAp translation
k_{2tm}	K_2in equation (2) for LacI-*lac* operon binding		

Table S5. Description of parameters in equations (3)-(5)

Type	Description	Type	Description
k_{1gm}	Equilibrium constant for TetR-TetO binding	k_{degR}	Degradation rate of TetR
k_{2gm}	Maximum rate of GFP mRNA production	τ_{mG}	Duration of GFP transcription
k_{3gm}	Dissociation constant for T7 RNAp-vector	τ_{pG}	Duration of GFP translation
k_{pG}	Production rate of GFP	τ_R	Duration of TetRproduction
k_{Gi}	Misfolding rate of GFP	n_{Gi}	Kinetic order of GFP misfolding
k_{1r}	Production rate of TetR via stress	n_R	Hill coefficient of TetRproduction
k_{degG}	Degradation rate of GFP		

Table S6. Description of parameters in equations (6)-(9).

Parameter	Value	Strain	Source
I_0	$1.00\ \mathrm{mM}$		Experimental
A	$8.10\ \mathrm{g\ L^{-1}h^{-1}}$		Estimated [1]
B	$1.64\ \times\ 10^{-1}\mathrm{g\ L^{-1}}$		Estimated [1]
k_{sat}	$2.00\ \times\ 10^{-2}\mathrm{h^{-1}}$	BL21	[1]
k_{m7}	$1.19\ \times\ 10^{-1}\mathrm{mM\ h^{-1}}$		Calculated
k_{1tm}	$1.00\ \times\ 10^{+1}\mathrm{mM^{-1}}$	RV80 & G141	[6]
k_{2tm}	$7.20\ \times\ 10^{+3}$	ML30	[2]
k_{p7}	$1.74\ \times\ 10^{+1}\mathrm{h^{-1}}$		Calculated
k_{1gm}	$7.39\ \times\ 10^{+5}\mathrm{mM^{-1}}$	RB 791	[7]
k_{2gm}	$3.12\ \times\ 10^{+3}\mathrm{h^{-1}}$	BL21	[8]
k_{3gm}	$1.50\ \times\ 10^{+1}\mathrm{mM}$	BL21	[8]
k_{pG}	$1.57\ \times\ 10^{+3}\mathrm{h^{-1}}$		Calculated
k_{Gi}	$2.98\ \times\ 10^{+2}\mathrm{h^{-1}mM^{-1.90}}$		Calculated
n_T	1.00	RV80 & G141	[6]
n_{Gi}	2.90		Estimated
τ_{m7}	$1.34\ \times\ 10^{-2}\mathrm{h}$	ML30	[2]
τ_{p7}	$1.16\ \times\ 10^{-2}\mathrm{h}$	ML30	[2]
τ_{mG}	$8.26\ \times\ 10^{-4}\mathrm{h}$	ML30	[2]
τ_{pG}	$3.31\ \times\ 10^{-3}\mathrm{h}$	ML30	[2]
k_{degM}	$8.00\ \mathrm{h^{-1}}$	NCM3416	[9]
k_{degT}	$8.40\ \times\ 10^{-1}\mathrm{h^{-1}}$		[10]
k_{degG}	$1.10\ \times\ 10^{-2}\mathrm{h^{-1}}$	BL21	Estimated [5]
k_{degR}	$3.60\ \times\ 10^{-2}\mathrm{h^{-1}}$	DH5α	[11]

Table S7. Parameters and rate constants used in the model along with the associated strain of *E. coli* they were obtained from, when applicable.

Parameter	Wild-type	Mutant, strong RBS	Mutant, weak RBS
k_{1r}	$9.50 \times 10^{-4}\mathrm{h}^{-1}\mathrm{mM}^{-0.41}$	$8.44 \times 10^{-4}\mathrm{h}^{-1}\mathrm{mM}^{-0.40}$	$1.30 \times 10^{-4}\mathrm{h}^{-1}\mathrm{mM}^{-0.90}$
τ_R	$8.30 \times 10^{-4}\mathrm{h}$	$9.02 \times 10^{-4}\mathrm{h}$	$4.83 \times 10^{-3}\mathrm{h}$
n_R	1.41	1.40	1.90

Table S8. Estimated parameters optimized for the three repression strengths.

Repression	$t = 2$ h	$t = 4$ h	$t = 6$ h	$t = 8$ h	$t = 10$ h
No feedback	7.66×10^{-9}	4.01×10^{-9}	1.28×10^{-7}	3.35×10^{-7}	7.66×10^{-9}
Wild-type	1.04×10^{-10}	5.78×10^{-9}	8.91×10^{-8}	3.33×10^{-7}	1.21×10^{-7}
Mutant, strong RBS	3.69×10^{-15}	3.69×10^{-15}	3.05×10^{-7}	8.11×10^{-9}	6.29×10^{-7}
Mutant, weak RBS	5.31×10^{-15}	5.31×10^{-15}	5.59×10^{-9}	9.36×10^{-8}	5.18×10^{-7}

Table S9. Squared error upon N-fold cross-validation (removal of one data point at the listed time).

4. Supplementary Figures

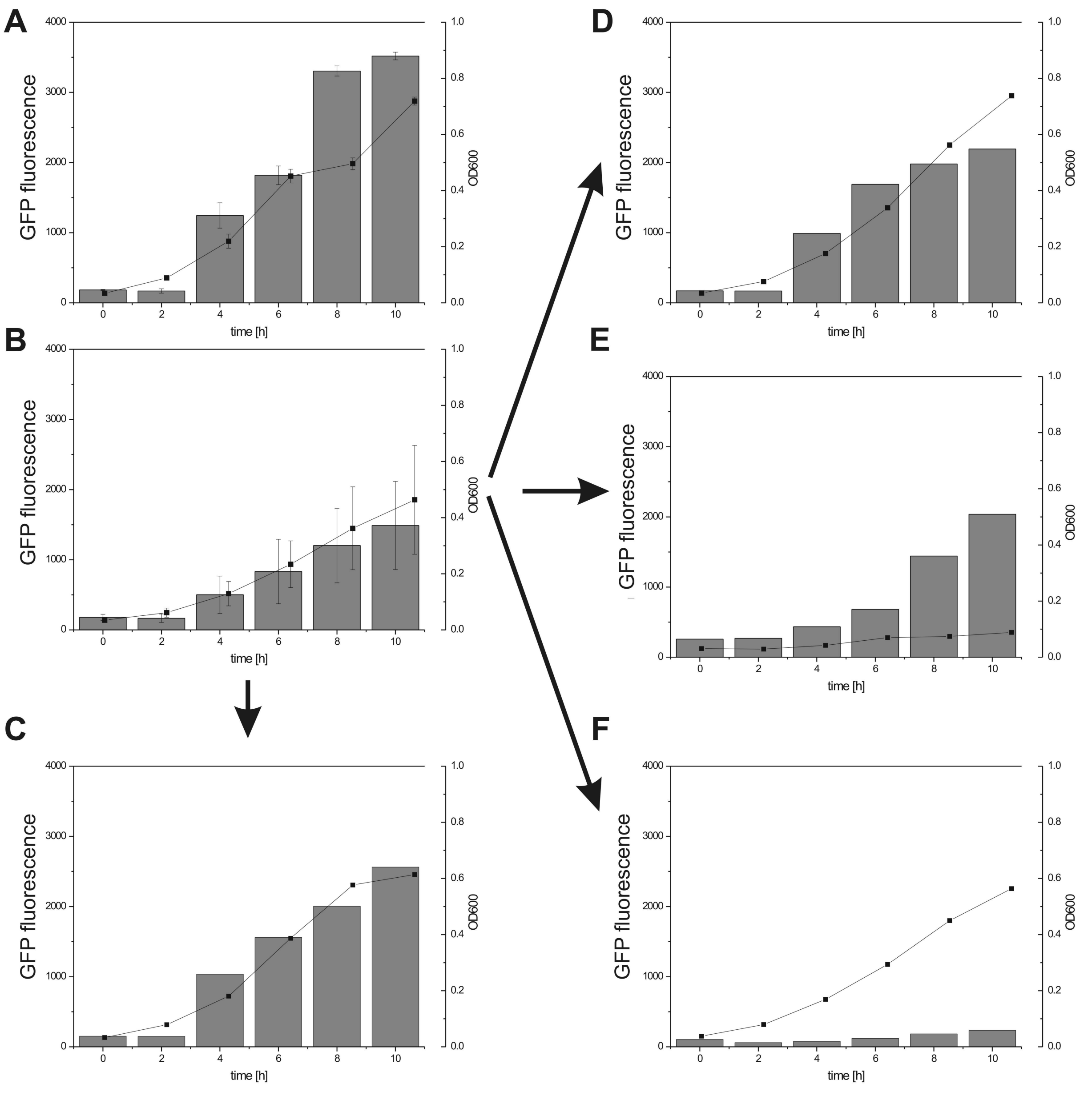

Figure S1. Variance in growth (lines) and GFP production (bars) in different clones of the initial pET23b_TetO / pNF_TetR (wildtype promoter strong RBS) system. (A) Average of the protein production system without feedback and with low variations between clones (B) wt P_{IbpAB} and strong RBS with high variation between clones (C-F) individual clones of wt P_{IbpAB} and strong RBS system.

WT PIbpAB

ATTCATCTGTTGATCGTGGGTGTTGGCCTGATGAGTTATAGCGATCCCTTGCTGAAAATAA
CATCATCATTACGTCGCACTGTGGCGGCTATCGCACTTTAACGTTTCGTGCTGCCCCCTC
AGTCTATGCAATAGACCATAAACTGCAAAAAAAAGTCCGCTGATAAGG**CTTGAA**AAGTTCA
TTTCCAG**ACCCATTT**TTACATC**G**TAGCCGAT

-10 -35

P_{IbpAB} Mutant 3_2

ATTCATCTGTTGATCGTGGGTGTTGGCCTGATGAGTTATAGCGATCCCTTGCTGAAA**G**TAA
CATCATCATTACGTCGCACTGTGGCGGCTATCGCACTTTAACG**C**TTCGTGC**T**GCCCCCTC
AGTCTATGCAATAGACCATAAACTGCAAAAAAAAGTCCGCTGATAAGG**CTTGAA**AAGTTCA
TTTCCAG**ACCCATTT**TTACATC**G**TAGCCGAT

-10 -35

PIbpAB Mutant 4_5

ATTCATCTGTTGATCGTGGGTGTTGGCCTGATGAGTTATAGCGATCCCTTGCTGAAAATAA
CATCATCATTACGTCGCACTGTGGCGGCTATCGCACTTTAACGTTTCGTGCTGCCCCCTC
AGTCTATGCAATAGACCATAAACTGCAAA**GC**AAAGTCC**C**CTGATAAGG**CTTGAA**AAGTTC
ATTTCCAG**ACCCATTT**TTACATC**G**TAGCCGAT

-10 -35

Figure S2. DNA sequences of different stress promoters based on the wildtype P_{IbpAB} that were used in this study. Bases highlighted in yellow/red indicate mutations compared with the wildtype promoter sequence.

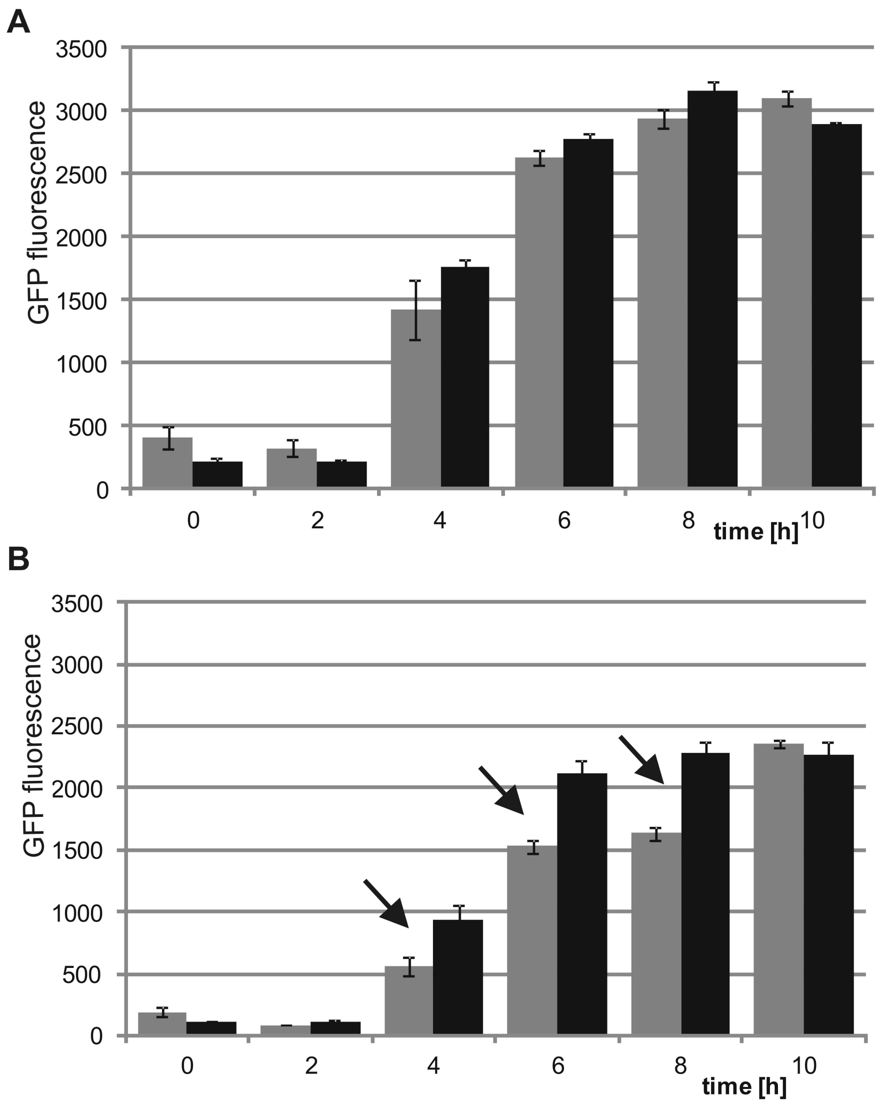

Figure S3. Starter culture growth medium influences an additional delay in GFP production in the system including the weak feedback (promoter mutant m4_5 and weak RBS). (A) starter cultures grown in M9 medium (0.4% glucose) and (B) starter cultures grown in LB medium. Grey bars – expression with feedback included and black bars represent GFP expression without feedback. Arrows in (B) highlight the additional delay. Data represent averages +/- standard error of the mean.

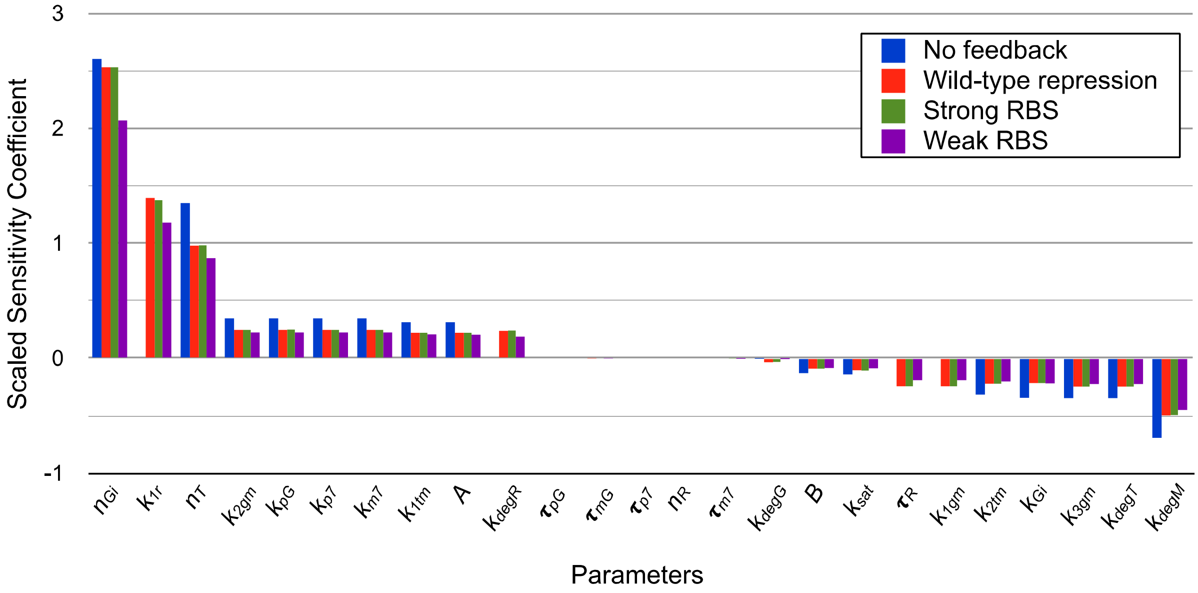

Figure S4. Sensitivity analysis. Scaled sensitivity coefficients reflect the influence of parameter perturbations on system response, using steady state GFP concentration as the readout.
